# A methodological review with meta-epidemiological analysis of preclinical systematic reviews with meta-analyses

**DOI:** 10.1038/s41598-022-24447-4

**Published:** 2022-11-21

**Authors:** Noémie Simon-Tillaux, Anne-Laure Gerard, Deivanes Rajendrabose, Florence Tubach, Agnès Dechartres

**Affiliations:** Sorbonne Université, INSERM, Institut Pierre Louis d’Epidémiologie et de Santé Publique, Département de Santé Publique, Centre de Pharmacoépidémiologie (Cephepi), Unité de Recherche Clinique PSL-CFX, CIC-1901, AP-HP, Hôpital Pitié Salpêtrière, 75013 Paris, France

**Keywords:** Medical research, Preclinical research

## Abstract

Systematic reviews and meta-analyses have been proposed as an approach to synthesize the literature and counteract the lack of power of small preclinical studies. We aimed to evaluate (1) the methodology of these reviews, (2) the methodological quality of the studies they included and (3) whether study methodological characteristics affect effect size. We searched MEDLINE to retrieve 212 systematic reviews with meta-analyses of preclinical studies published from January, 2018 to March, 2020. Less than 15% explored the grey literature. Selection, data extraction and risk of bias assessment were performed in duplicate in less than two thirds of reviews. Most of them assessed the methodological quality of included studies and reported the meta-analysis model. The risk of bias of included studies was mostly rated unclear. In meta-epidemiological analysis, none of the study methodological characteristics was associated with effect size. The methodological characteristics of systematic reviews with meta-analyses of recently published preclinical studies seem to have improved as compared with previous assessments, but the methodological quality of included studies remains poor, thus limiting the validity of their results. Our meta-epidemiological analysis did not show any evidence of a potential association between methodological characteristics of included studies and effect size.

## Introduction

The median cost to develop a new drug is now more than half a billion dollars^1^. A prominent part of this bill is related to preclinical research, in which animal model-based and bench studies are conducted to test drug efficacy and safety. These studies are a mandatory step in the drug development process because exposing patients to a compound without a proof-of-concept of its potential interest would be unethical. However, even if promising results are obtained in the setting of preclinical research, most of the tested molecules fail to translate their efficacy in clinical trials^2^. In addition, concerns have been raised by the research community about the poor reproducibility of results, which leads them to question the methodological quality and the reporting of preclinical research^3^. Indeed, most preclinical studies are underpowered, with no sample size calculation prior to conducting experiments, which increases the risk of false conclusions^4^. As well, randomization and blinding, which are key methodological elements for causal inference, are usually not performed, which increases the risk of selection, performance and measurement biases^5^. Attrition bias may be another important issue. Removing outliers from the final analysis or not reporting dead animals without a measurable outcome seem common practice and may completely change the direction and magnitude of effect size^6^. Finally, reporting bias may be problematic because small studies with a striking effect are more likely to be published than those with negative results^7^.

In the context of small-sample studies, systematic reviews and meta-analyses seem attractive to synthesize results and overcome the lack of power^8^. Some previous studies suggested that systematic reviews of preclinical studies may have poor methodology, which led the Collaborative Approach to Meta-Analysis and Review of Animal Data from Experimental Studies (CAMARADES) and the Systematic Review Centre for Laboratory animal Experimentation (SYRCLE) in the early 2010s to provide guidance to conduct systematic reviews in the specific context of preclinical research^9–14^. Since the publication of these recommendations, the methodological quality of these reviews has not been evaluated, nor has the impact of methodological features of the included studies on the effect size.

In this study, we aimed to evaluate (1) the methodology of recently published systematic reviews with meta-analyses of preclinical research; (2) the methodological quality of preclinical studies included in such reviews and (3) the association between methodological characteristics of the included studies and effect size by using a meta-epidemiological approach.

## Results

### Selection and general characteristics of systematic reviews with meta-analyses of preclinical studies

**Figure 1:**
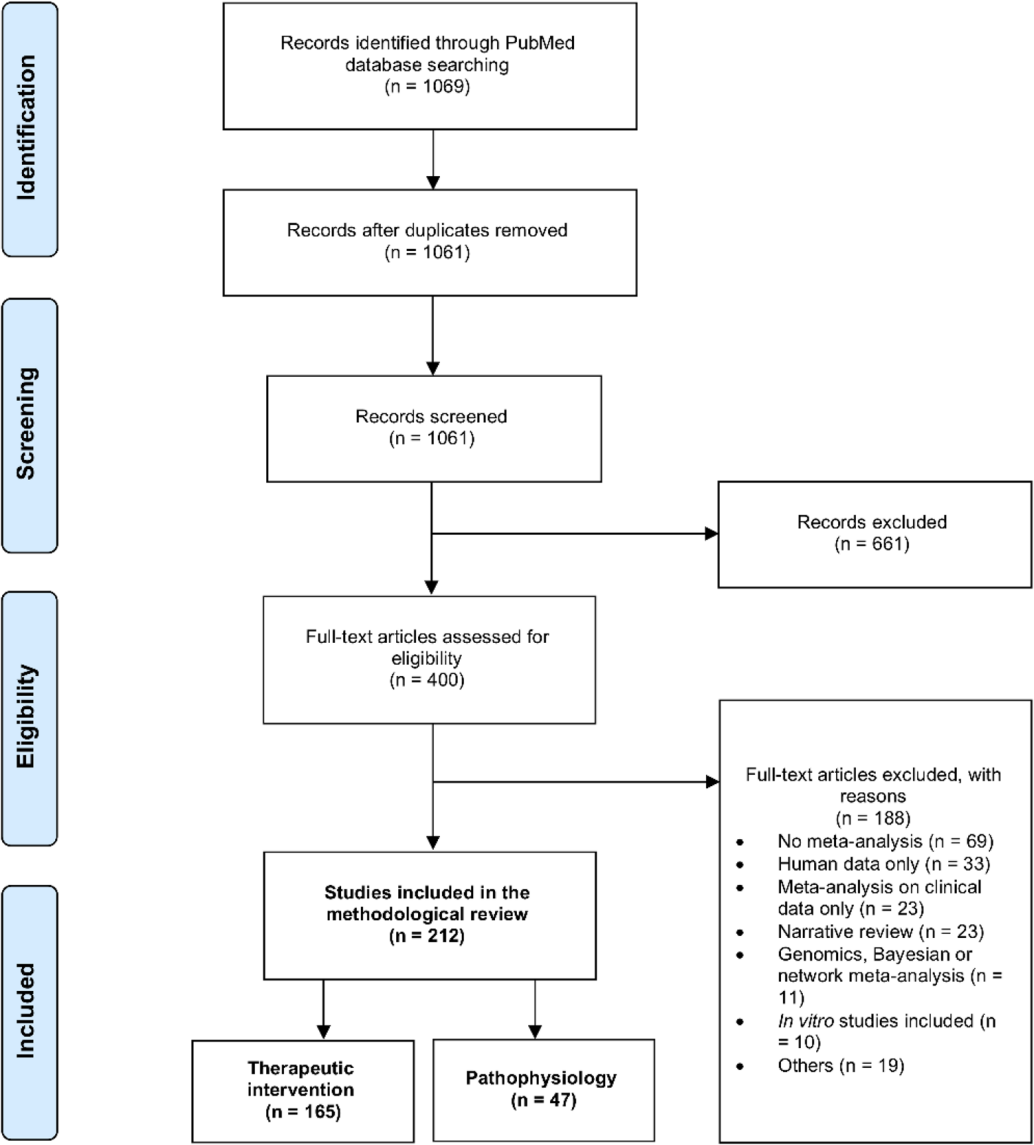
Selection process of preclinical systematic reviews with meta-analyses. Adapted from the PRISMA flow diagram^18^.

Our search retrieved 1061 unique reviews. After sorting them based on eligibility criteria, 212 systematic reviews with meta-analyses were included in this methodological review (Fig. 1, and Supplementary File S2 for the complete list of included reviews).

General characteristics of these reviews are presented in Table 1. The medical conditions studied were most commonly related to neurology or neurosurgery (27.8% of reviews). A statistician or an epidemiologist was involved in 22.2% of the articles. Overall, 75.9% of the reviews reported adherence to guidelines for conducting the systematic review: the PRISMA Statement reported in 86.3%, and guidelines from SYRCLE and CAMARADES consortia followed in 20.5% and 3.1%, respectively. A low number of reviews were registered (25.0%), mainly on the PROSPERO registry or on SYRCLE or CAMARADES website. Regarding the definition of the objective, the population and intervention were well reported in most systematic reviews (97.2% and 97.6%, respectively), but the control and outcome (with timepoint) were clearly defined in only 59% and 34% (Supplementary Table S1).

**Table 1:** General characteristics of the included systematic reviews with meta-analyses. Adapted from the PRISMA flow diagram^18^.

	Overall	Therapeutic intervention	Pathophysiology
	(N = 212)	(N = 165)	(N = 47)
**Medical field**			
Neurology/neurosurgery	59 (27.8%)	50 (30.3%)	9 (19.1%)
Dentistry	23 (10.8%)	19 (11.5%)	4 (8.5%)
Cardiology/cardiac surgery	17 (8.0%)	13 (7.9%)	4 (8.5%)
Endocrinology/nutrition	15 (7.1%)	8 (4.8%)	7 (14.9%)
Rheumatology	12 (5.7%)	1 (0.6%)	11 (23.4%)
Toxicology	10 (4.7%)	10 (6.1%)	0 (0%)
Respiratory medicine	10 (4.7%)	9 (5.5%)	1 (2.1%)
Gastroenterology/hepatology	8 (3.8%)	8 (4.8%)	0 (0%)
Infectious diseases	9 (4.2%)	8 (4.8%)	1 (2.1%)
Oncology	9 (4.2%)	6 (3.6%)	3 (6.4%)
Psychiatry	9 (4.2%)	7 (4.2%)	2 (4.3%)
Medicine (others)	16 (7.5%)	11 (6.7%)	5 (10.6%)
Surgery (others)	15 (7.1%)	15 (9.1%)	0 (0%)
**Statistician or epidemiologist involved**	47 (22.2%)	32 (19.4%)	15 (31.9%)
**Funding source**			
Public/non-profit organization	135 (63.7%)	104 (63.0%)	31 (66.0%)
Public and private	7 (3.3%)	6 (3.6%)	1 (2.1%)
Private	1 (0.5%)	0 (0%)	1 (2.1%)
No funding	30 (14.2%)	25 (15.2%)	5 (10.6%)
Not reported	39 (18.4%)	30 (18.2%)	9 (19.1%)
**Conflict of interest**			
Yes	18 (8.5%)	14 (8.5%)	4 (8.5%)
No	170 (80.2%)	139 (84.2%)	31 (66.0%)
Not reported	24 (11.3%)	12 (7.3%)	12 (25.5%)
**Guidelines**			
Yes	161 (75.9%)	126 (76.4%)	35 (74.5%)
*PRISMA statement*	139 (86.3%)	109 (86.5%)	30 (85.7%)
*Cochrane Handbook*	16 (9.9%)	14 (11.1%)	2 (5.7%)
*SYRCLE guidelines*	33 (20.5%)	24 (19.0%)	9 (25.7%)
*CAMARADES guidelines*	5 (3.1%)	5 (4.0%)	0 (0%)
*Other guidelines*	5 (3.1%)	3 (2.4%)	2 (5.7%)
Not reported	51 (24.1%)	39 (23.6%)	12 (25.5%)
**Registration of protocol**			
Yes	53 (25.0%)	43 (26.1%)	10 (21.3%)
*PROSPERO registry*	23 (43.4%)	17 (39.5%)	6 (60.0%)
*SYRCLE or CAMARADES website*	24 (45.3%)	19 (44.2%)	5 (50.0%)
*Others*	8 (15.1%)	8 (18.6%)	0 (0%)
No	159 (75.0%)	122 (73.9%)	37 (78.7%)
**Characteristics of journals**			
Peer-reviewed journal	212 (100%)	165 (100%)	47 (100%)
IF of journal, median [IQR]	3.72 [1.06, 12.0]	3.72 [1.06, 12.0]	3.85 [1.34, 12.0]

### Methodological characteristics of systematic reviews with meta-analyses of preclinical studies

All reviews conducted an electronic search, and almost all (99.1%) reported at least one electronic database. The search equation was reported in 66.5% of reviews, and an animal filter was applied in 10.8%. A search of other sources was reported for 62.7% of reviews, predominantly the exploration of reference list of selected studies (75.9%). The grey literature was explored in 25 (11.8%) reviews and the OpenGrey website for 6 (4.5%). The selection and data extraction process were performed in duplicate in 67.9% and 46.7% of reviews, respectively. Methodological quality of included studies was assessed in 83.5% of reviews and in duplicate in 59.3%. Reviews of pathophysiologic studies less often assessed the methodological quality (70.2% vs 87.3% of reviews of therapeutic interventions). The most common tools used were the SYRCLE risk of bias tool (50.8%) and the CAMARADES quality checklist (18.6%) (Table 2).

**Table 2:** Methodological characteristics of included systematic reviews. *Chinese National Knowledge Infrastructure, WanFang, VIP, Chinese Biomedical Literature. **As published by Hooijmans et al., and de Vries et al.^47,50^. ***Google search engine queries, OpenGrey website, experts, unpublished data, others websites.

	Overall	Therapeutic intervention	Pathophysiology
	(n = 212)	(n = 165)	(n = 47)
**Search strategy**			
Electronic search	210 (99.1%)	164 (99.4%)	46 (97.9%)
*Medline*	209 (99.5%)	163 (99.4%)	46 (100%)
*Embase*	135 (64.3%)	106 (64.6%)	29 (63.0%)
*Scopus*	43 (20.5%)	33 (20.1%)	10 (21.7%)
*Web of science*	91 (43.3%)	69 (42.1%)	22 (47.8%)
*Cochrane library*	57 (27.1%)	49 (29.9%)	8 (17.4%)
*Google Scholar*	32 (15.2%)	24 (14.6%)	8 (17.4%)
Chinese language databases*	37 (17.6%)	33 (20.1%)	4 (8.7%)
*Other databases*	71 (33.8%)	54 (32.9%)	17 (37.0%)
No database reported	2 (0.9%)	1 (0.6%)	1 (2.1%)
Reported search equation			
Yes	141 (66.5%)	106 (64.2%)	35 (74.5%)
Only keywords or unclear equation	64 (30.2%)	54 (32.7%)	10 (21.3%)
Use of an animal filter**	23 (10.8%)	17 (10.3%)	6 (12.8%)
Other sources of identification of studies	133 (62.7%)	104 (63.0%)	29 (61.7%)
*Reference list of selected studies*	101 (75.9%)	79 (76.0%)	22 (75.9%)
*Reference list of other relevant publications (reviews, comments)*	57 (42.9%)	47 (45.2%)	10 (34.5%)
*Abstract of conferences, specific journals*	16 (12.0%)	15 (14.4%)	1 (3.4%)
Grey literature***	25 (11.8%)	14 (8.5%)	11 (23.4%)
**Selection process**			
Definition of eligibility criteria	208 (98.1%)	162 (98.2%)	46 (97.9%)
Restriction of language	110 (51.9%)	85 (51.5%)	25 (53.2%)
Restriction of publication date	34 (16.0%)	23 (13.9%)	11 (23.4%)
Selection in duplicate	144 (67.9%)	112 (67.9%)	32 (68.1%)
**Data extraction process**			
Data extraction in duplicate	99 (46.7%)	82 (49.7%)	17 (36.2%)
**Methodological quality**			
Assessment of risk of bias/methodological quality	177 (83.5%)	144 (87.3%)	33 (70.2%)
*SYRCLE risk of bias*	90 (50.8%)	72 (50.0%)	18 (54.5%)
*CAMARADES quality checklist*	33 (18.6%)	30 (20.8%)	3 (9.1%)
*Cochrane risk of bias tool*	19 (10.7%)	17 (11.8%)	2 (6.1%)
*Quality of reporting ARRIVE*	21 (11.9%)	16 (11.1%)	5 (15.2%)
*Risk of bias STAIR*	14 (7.9%)	12 (8.3%)	2 (6.1%)
*Risk of bias OHAT*	5 (2.8%)	1 (0.7%)	4 (12.1%)
*Other assessments of risk of bias*	20 (11.3%)	16 (11.1%)	4 (12.1%)
Methodological quality assessment in duplicate	105 (59.3%)	18 (54.5%)	87 (60.4%)

The median number of included studies per systematic review was 22 (interquartile range [Q1, Q3] 13, 42), with a trend to a higher number in systematic reviews exploring the pathophysiology of diseases (27 [15–62]). The animal model (species and strain) was detailed in 59.0% of reviews.

The methodological characteristics of the meta-analyses are summarized in Table 3. Quantitative outcomes were mostly studied (92.5%) and were summarized with an SMD (61.7%). The random-effects model was the most frequently used (84.5%), and 29.0% of authors advocated for this statistical model based on an observed substantial statistical heterogeneity. Only 9 reviews used both fixed- and random-effects models to synthesize data. Almost two thirds of meta-analyses (61.8%) included several experimental arms for the same preclinical study; 21.2% used a method to take into account this particularity (splitting the control group according to the number of experimental arms sharing the control^10^, robust variance-based or multilevel models^15,16^). The statistical heterogeneity was measured in 93.4% of meta-analyses by the Cochran Q test and/or the I^2^ and was explored in 63.7%. The impact of the methodological quality on meta-analysis results was assessed in 11.3% of meta-analyses. Small-study effects were explored in 64.5% of meta-analyses including 10 experimental arms or more. In total, 33 (15.6%) meta-analyses included both animal and human studies in their systematic review. More than half (54.5%; n = 18) performed separate meta-analyses according to human or animal data, and 27.3% (n = 9) meta-analyzed only animal data (Supplementary Table S2).

**Table 3:** Methodological characteristics of the meta-analyses (for the primary outcome or first reported outcome). *MA* meta-analysis. *Splitting the control group according to the number of experimental arms, use of robust variance in meta-analysis model, multi-level random-effects models. **Egger’s or Begg’s tests, other tests.

	Overall	Therapeutic intervention	Pathophysiology
	(n = 212)	(n = 165)	(n = 47)
**Class of outcome and effect size**			
Categorical outcome	14 (6.6%)	13 (7.9%)	1 (2.1%)
*Risk ratio*	10 (71.4%)	9 (69.2%)	1 (100%)
*Odds ratio*	4 (28.6%)	4 (30.8%)	0 (0%)
Quantitative outcome	196 (92.5%)	150 (90.9%)	46 (97.9%)
*Standardized mean difference*	121 (61.7%)	90 (60.0%)	31 (67.4%)
*Mean difference*	62 (31.6%)	51 (34.0%)	11 (23.9%)
*Normalized mean difference*	4 (2.0%)	3 (2.0%)	1 (2.2%)
*Others*	9 (4.6%)	6 (4.0%)	3 (6.5%)
Time-to-event (hazard ratio)	1 (6.2%)	1 (6.7%)	0 (0%)
**MA model reported**			
Random-effects model	175 (84.5%)	134 (83.8%)	41 (87.2%)
Fixed-effect model	32 (15.5%)	30 (18.8%)	2 (4.3%)
Fixed-effect or random-effects model according to observed I^2^	60 (29.0%)	52 (32.5%)	8 (17.0%)
Use of both fixed-effect and random-effects models	9 (4.3%)	8 (5.0%)	1 (2.1%)
Other models (multi-level models, use of robust variance)	11 (5.3%)	5 (3.1%)	6 (12.8%)
Not reported	5 (2.4%)	5 (3.0%)	0 (0%)
**Several experimental arms of the same study included in the MA**	131 (61.8%)	97 (58.8%)	34 (72.3%)
If yes, adjustment for several experimental arms of the study*	45 (21.2%)	34 (20.6%)	11 (23.4%)
**Assessment of statistical heterogeneity (Cochrane Q test, I**^2^**)**	198 (93.4%)	159 (96.4%)	39 (83.0%)
**Exploration of heterogeneity**			
Yes	135 (63.7%)	105 (63.6%)	30 (63.8%)
*Subgroup analysis*	108 (80.0%)	89 (84.8%)	19 (63.3%)
*Meta-regression*	49 (36.3%)	31 (29.5%)	18 (60.0%)
*Others: sensitivity analysis without outliers, leave-one-out analysis*	14 (10.4%)	11 (10.5%)	3 (10.0%)
No	67 (31.6%)	55 (33.3%)	12 (25.5%)
**Impact of study methodological quality on MA results**			
Yes	24 (11.3%)	19 (11.5%)	5 (10.6%)
Sensitivity analysis without high-risk bias studies	4 (16.7%)	4 (21.1%)	0 (0%)
Subgroup analysis by risk of bias	5 (20.8%)	3 (15.8%)	2 (40.0%)
Subgroup analysis on blinding of assessors	7 (29.2%)	6 (31.6%)	1 (20.0%)
Meta-regression on quality score	7 (29.2%)	5 (26.3%)	2 (40.0%)
Others	5 (20.8%)	3 (15.8%)	2 (40.0%)
No	154 (72.6%)	125 (75.8%)	29 (61.7%)
**Meta-analyses with ≥ 10 experimental arms**	138 (65.0%)	104 (63.0%)	33 (70.2%)
Assessment of small-study effects	89 (64.5%)	73 (70.2%)	16 (48.5%)
Tests for funnel plot asymmetry**	66 (47.8%)	51 (49.0%)	15 (45.5%)
Trim-and-fill analysis	31 (22.5%)	23 (22.1%)	8 (24.2%)

The median number of included studies in the meta-analyses was 9 [Q1, Q3 5, 20.0] and median number of experimental arms 13 [7, 33.5] (Supplementary Table S3). Heterogeneity was generally high across studies, with a median I^2^ of 77% [55.5, 90.7]. Small study effects were reported in 35.5% of meta-analyses that included ≥ 10 experimental arms and reported this evaluation. Among the 121 reviews that used an SMD, the combined estimates were in favor of the treatment (or the expected direction for reviews of pathophysiology studies) with a p-value below 5% for 104 (86.6%), not associated with the treatment for 6, and not in favor of the treatment for 1 with a p-value below 5%; in 10 meta-analyses, the expected direction of the effect size was unclear.

### Methodological features of included preclinical studies

We evaluated 763 unique studies from 63 meta-analyses reporting an evaluation of methodological quality for each study and an SMD as the effect size (see Supplementary Fig. S2 for details on the selection process). The median year of publication was 2013 [Q1, Q3: 2009, 2015]; 44.0% of studies originated from a Chinese laboratory, and 78.9% were published in English. Most experimental procedures (92.7%) involved rodents (Table 4).

**Table 4:** General characteristics of the included studies in meta-analyses.

General characteristics	Overall
	(N = 763)
**Year of publication, median [Q1, Q3]**	2013 [2009, 2015]
**Country of laboratory**	743 (97.4%)
China	327 (44.0%)
USA	104 (14.0%)
Japan	51 (6.9%)
Republic of Korea	47 (6.3%)
Canada	23 (3.1%)
Brazil	21 (2.8%)
Others	170 (22.9%)
**Language of publication**	741 (97.1%)
English	585 (78.9%)
Chinese	154 (20.8%)
Others	2 (0.3%)
**Animal model**	751 (98.4%)
Rodents	696 (92.7%)
Pigs	18 (2.4%)
Dogs	20 (2.7%)
Sheep	6 (0.8%)
Other animal models	11 (1.5%)
**Scales for quality evaluation**	826 (100.0%)
SYRCLE risk of bias tool	304 (39.8%)
CAMARADES quality checklist	172 (22.5%)
Cochrane Risk of Bias tool	34 (4.5%)
STAIR recommendations checklist	48 (6.3%)
OHAT risk of bias rating tool	29 (3.8%)
Other scales	246 (32.2%)

Among items from the different scales used to appraise risk of bias (Table 4), randomization was rated for all studies (Table 5). However, blinding of the animal model was available for only 25.4% of included studies and was mainly rated at high risk (92.3%). The following characteristics were mostly rated at unclear risk of bias: allocation concealment (60.7%), random housing of animals (91%), blinding of caregivers (77.8%), random outcome assessment (86.3%) and blinding of assessors (39.8%). Low risk was predominantly reported for randomization (53%), similarity of group characteristics at baseline (51.9%), attrition bias (60.3%), and selective outcome reporting bias (68.3%).

**Table 5:** Risk of bias of included studies in the meta-analyses.

Risk of bias items	Overall
	(N = 763)
**Randomization**	763 (100.0%)
High risk	130 (17.0%)
Low risk	406 (53.2%)
Unclear	227 (29.8%)
**Group characteristics similar at baseline**	283 (37.1%)
High risk	2 (0.8%)
Low risk	147 (51.9%)
Unclear	134 (47.3%)
**Respect of allocation concealment**	425 (55.7%)
High risk	102 (24.0%)
Low risk	65 (15.3%)
Unclear	258 (60.7%)
**Random housing of animals**	256 (33.6%)
High risk	12 (4.7%)
Low risk	11 (4.3%)
Unclear	233 (91.0%)
**Blinding of caregivers**	316 (41.4%)
High risk	53 (16.8%)
Low risk	17 (5.4%)
Unclear	246 (77.8%)
**Blinding of animal model**	194 (25.4%)
High risk	179 (92.3%)
Low risk	15 (7.7%)
Unclear	0 (0%)
**Random outcome assessment**	256 (33.6%)
High risk	17 (6.6%)
Low risk	18 (7.0%)
Unclear	221 (86.3%)
**Blinding of assessors**	603 (79.0%)
High risk	180 (29.9%)
Low risk	183 (30.3%)
Unclear	240 (39.8%)
**Attrition**	514 (67.4%)
High risk	73 (14.2%)
Low risk	310 (60.3%)
Unclear	131 (25.5%)
**Selective outcome reporting**	322 (42.2%)
High risk	24 (7.5%)
Low risk	220 (68.3%)
Unclear	78 (24.2%)

We also assessed items regarding the quality of reporting and methods of experimental procedures. The number of studies evaluated for a given feature by review authors is reported in Table 6. “Yes” was assigned in half or more of studies assessed for the features publication in a peer-reviewed journal, control of temperature during the experimental procedure, use of anesthetic without protective effect on outcome, description of animal model, compliance with animal welfare regulations and statement of conflict of interest. However, sample size calculation was reported in only 1 of 351 studies assessed for this feature.

**Table 6:** Quality of reporting and experimental procedures of included studies in meta-analyses.

Reporting characteristics	Overall
	(N = 763)
**Publication in a peer-reviewed journal**	293 (38.4%)
Yes	257 (87.7%)
No	36 (12.3%)
**Control of temperature**	275 (36.0%)
Yes	165 (60.0%)
No	110 (40.0%)
**Use of anesthetic without protective effect on outcome**	141 (18.5%)
Yes	110 (78.0%)
No	31 (22.0%)
**Description of animal model**	256 (33.6%)
Yes	141 (55.1%)
No	115 (44.9%)
**Sample size calculation**	351 (46.0%)
Yes	1 (0.3%)
No	350 (99.7%)
**Compliance with animal welfare regulations**	399 (52.3%)
Yes	258 (64.7%)
No	141 (35.3%)
**Statement of conflict of interest**	284 (37.2%)
Yes	142 (50.0%)
No	142 (50.0%)

### Meta-epidemiological analysis

**Figure 2:**
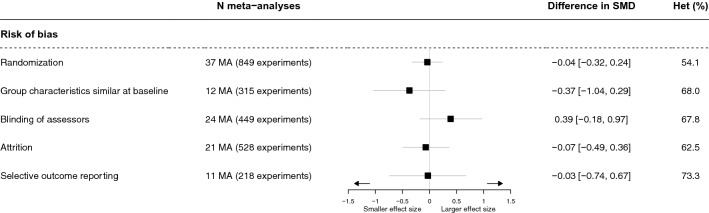
Difference in standardized mean difference (SMD) for risk of bias estimated by a meta-epidemiological analysis. A positive difference in SMD reveals a larger effect size in studies at high or unclear risk of bias. A negative difference in SMD indicates a smaller effect size in case of threats to methodological quality. *Het* heterogeneity, *MA* meta-analyses.

Among the 63 selected meta-analyses, the number contributing to the analysis for each key methodological feature ranged from 11 (218 experimental arms) to 37 (849 experimental arms) (Fig. 2). None of the risk of bias features that could be evaluated (randomization, group characteristics similar at baseline, blinding of assessors, attrition, and selective outcome reporting) was associated with effect size. The I^2^ ranged from 54.1% (randomization) to 73.3% (selective outcome reporting). The sensitivity analysis using a robust variance estimator gave similar results (Supplementary Fig. S3).

We did not perform the meta-epidemiological analysis for allocation concealment, random housing of animals, blinding of caregivers and animal models, and random outcome assessment, because of fewer than ten eligible meta-analyses for these characteristics (Supplementary Table S4).

## Discussion

This review provides an overview of the methodological quality of recently published systematic reviews with meta-analyses of preclinical research. Regarding the systematic review process, an electronic database was almost always reported, and most systematic reviews assessed the methodological quality of included studies. In addition, almost all reviews reported the meta-analysis model and an evaluation of statistical heterogeneity, which was explored in two thirds of reviews. Still, there is some room for improvement because a clear definition of the control group was often missing both in the methods and in the description of included studies in results. Selection, data extraction and risk of bias assessment were performed in duplicate in only 67.9%, 46.7% and 59.3% of reviews, respectively. In addition, the methodological quality of included studies was poor: the risk of bias of included studies was mostly rated unclear, and reporting was often incomplete. Our meta-epidemiological analysis did not find any potential association of a methodological feature with the study effect size.

As compared with previously published methodological reviews, our evaluation suggests a substantial improvement in the methodology of systematic reviews and meta-analyses of preclinical studies. Across all evaluated reviews, the PRISMA statement was followed in 65.5% of reports, which contrasts with the previous reported rates of 3–14%^9,10,13,17–19^. Also, our study revealed a much more frequent evaluation of the methodological quality of the included studies (83.5% vs 18–47.3% in previous reviews^11–13,20,21^). This improvement might reflect the positive impact of the 2014 publication of the SYRCLE risk of bias tool, whose development in line with the Cochrane Risk of Bias tool may have facilitated its quick adoption in systematic reviews of preclinical research^22–24^. The methodological features related to meta-analysis also showed improvement: heterogeneity was assessed in 93.4% of meta-analyses and the small-study effect was explored in two thirds of reviews as compared with 19% and 22%, respectively, in previous published methodological reviews^11–13,21^.

Some of our results are consistent with an evaluation of systematic reviews and meta-analyses of clinical studies^25^. Similar to meta-analyses of clinical data, for preclinical data, we found a low rate of searching for grey literature; an assessment of risk of bias in most reviews but seldom taken into account in the meta-analysis; and an inadequate use of the heterogeneity statistic to guide the choice of model in about one third of meta-analyses, which is not recommended by Cochrane^26^. The two main differences from meta-analyses of clinical studies were the type of primary outcome, mostly continuous in our sample of preclinical reviews and dichotomous in clinical research reviews, and the number of effect estimates that could be some possible associations. Indeed, Page et al. found that 60% of meta-analyses combined estimates were different from a null effect considering a type-one error rate below 5% as compared with 86.6% of meta-analyses of SMD in our sample^25^.

Regarding preclinical studies, our results suggest an improvement in some domains but not in others, which is consistent with previous published methodological reviews. Van Lujk et al. reported the quality of studies included in 91 systematic reviews (published between 2005 and 2012) of interventions in animals and found similar rates of blinding of caretakers, assessors, and drop-outs in primary studies^20^. Conversely, we found a better reporting of randomization (53.2%), which is consistent with a recently published methodological review^27^, and of conflicts of interest (50.0%), which confirms the time-trend observed in the review of preclinical studies by Macleod et al. for these methodological features^4^. However, the mere reporting of randomization should be balanced by its evaluation with the more stringent SYRCLE risk of bias item “appropriate sequence generation”: the rate of low-risk studies then decreases to 33.5% (130/388). In the same way, even if features such as selective outcome reporting and attrition were considered predominantly at low risk by review authors, the registration of the protocol of preclinical studies and presenting a flow chart are still uncommon, which limits the evaluation of these biases. Of note, random housing of animals and random outcome assessment were almost always graded as unclear, which might reflect the lack of these practices in laboratories. Sample size calculation before starting the experimental procedure was almost never reported, which is consistent with a previous report pointing to the stability over time of poor use of statistics in preclinical research^4,27^. Some studies have evaluated the impact of reporting guidelines on quality of studies submitted to journals^28,29^. Unfortunately, contrary to dedicated guidelines for clinical studies such as the CONSORT statement^30^, the 2010 implementation of the ARRIVE checklist^31,32^ in the submission process did not seem to affect quality of reporting^29^. This finding underlines the importance of a multi-dimensional response to this issue: endorsement of guidelines by institutions and publishers and guaranteeing that they are actually followed could help improve the quality of preclinical research^33,34^. A better awareness of the importance of methodology and transparency in research at all stages of training may also help change practices.

Our meta-epidemiological analysis failed to show any possible association between the methodological characteristics of included studies and the effect size. However, our analysis may have been underpowered in that numerous systematic reviews assigned to all studies the same rating for a given methodological feature, which prevents a meta-epidemiological analysis. Particularly, analysis was precluded with risk of bias items mostly rated as unclear as a reflection of poor reporting of preclinical studies. The analysis may also have been dogged by substantial heterogeneity between meta-analyses, with I^2^ > 50% for all characteristics evaluated. This statistical heterogeneity may reveal variability in rating of the methodological quality across the reviews. In addition, these results may be explained by a differential impact of methodological features on the effect size between clinical and preclinical research. For example, meta-epidemiological approaches of clinical studies consistently reported an association between inadequate sequence generation and larger treatment effect^35–38^. Our meta-epidemiological analysis of preclinical data might have failed to demonstrate this association because in preclinical studies, the experiments are often conducted in inbred strains, which limits the between-subject variability and accordingly the impact of a biased randomization.

Few meta-epidemiological analyses have examined preclinical studies, but their results are consistent with our own. Crossley et al. used a similar two-step meta-epidemiological approach to study the impact of randomization, blinding of the animal model and blinding of assessors on 13 meta-analyses of experimental stroke studies; except for blinding of the model, none of these features was associated with the effect size, but only 7 meta-analyses were included in the analysis of blinding^39–45^. Another study evaluated the impact on the effect size of blinding of outcome assessment in experimental spinal cord injury by estimating the proportion of variance explained by the methodological feature. The study found a very low value associated with this characteristic^46^.

Our study has several strengths: for the first time we assessed the methodological characteristics of systematic reviews with meta-analyses of preclinical research in light of dedicated recommendations, based on a large sample of recently published reviews and concomitantly assessed the methodological quality of the studies they included. We also performed a meta-epidemiological analysis of a wide range of methodological features to evaluate how these characteristics could modify the reported effect sizes. Notably, we did not limit our assessment to studies related to neurological diseases as in previous published meta-epidemiological analyses. Nevertheless, our study presents several limitations. Our search strategy might have induced a selection bias because we searched only the MEDLINE database and included only systematic reviews associated with meta-analyses, which may feature greater methodological quality than the overall literature^11–13^. Our meta-epidemiological analysis also has two weaknesses: lack of power as discussed above and the heterogeneity of results, which may be explained by differences in rating between review authors in that we relied on only data extracted from systematic reviews.

To conclude, even if the overall methodological profile of systematic reviews with meta-analyses of preclinical research seems to show substantial improvement, the reporting and methodological quality of the studies they include may still jeopardize their internal validity and limit their interpretation to exploratory data. Our meta-epidemiological analysis did not reveal any possible association between the methodological characteristics of included studies and the effect size, but it may have been underpowered. Also, meta-epidemiological studies are observational by nature and may be affected by the quality of reporting in included studies. Therefore, these results should not be used as an excuse to not comply with the well-established guidelines for conducting experimental studies. Institutions and publishers have endeavored to improve the overall methodological quality of preclinical data, but there is still a long way to go before animal studies reach expected methodological standards.

## Methods

This is a methodological review of systematic reviews with meta-analyses of preclinical studies with a meta-epidemiological analysis of study characteristics associated with effect size. A protocol (available upon request) was written before starting the review process.

### Search strategy

We searched MEDLINE via PubMed for systematic reviews with meta-analyses of preclinical studies published between January 1, 2018 and March 1, 2020. We focused on MEDLINE because it is the most widely used database and because our aim was not to be exhaustive but to retrieve a large sample of recent studies. The search algorithm combined terms for meta-analyses and preclinical, in vivo or animal studies and their synonyms^47^ (Supplementary File S1).

### Eligibility criteria

We selected all systematic reviews with meta-analyses reporting the evaluation of a pharmacological (i.e., any administered product with biological activity) or non-pharmacological intervention to treat one or several medical conditions or to explore the pathophysiology of a disease. Only meta-analyses published in English were included. All vertebrate animal models were eligible, regardless of age and sex. If the review aimed to summarize preclinical and clinical evidence, we selected only articles in which the meta-analysis section included preclinical studies.

The following studies were excluded: systematic reviews without meta-analysis; systematic reviews and meta-analyses without an evaluation of an intervention (e.g., evaluation of performance of a diagnostic test); systematic reviews including in vitro experimentation about ecology, veterinary medicine, agri-food industry, microorganism epidemiology, microbiology, and human-only research (including genetics or medical biochemistry studies); reviews of invertebrate models; narrative reviews; methodological studies; case reports; editorials; protocols; replication studies and comments; network and Bayesian meta-analyses; and meta-analyses of transcriptomic, genomic or epigenetic studies^48^.

### Selection process

References retrieved by the search were imported to a reference manager (Zotero 5.0). The title and abstract were first screened by one reviewer (NST). The full text was assessed for eligibility by two reviewers (NST and ALG). Disagreements were solved by consensus.

### Data extraction

Two reviewers (NST and DR) extracted data for all selected studies according to two standardized data extraction forms (one for systematic reviews and meta-analyses and one for included preclinical studies). Disagreements were resolved by consensus.

#### Characteristics of systematic reviews and meta-analyses

From available recommendations for systematic reviews and meta-analyses of preclinical studies^9,10,14^, we extracted the following data for each systematic review.

General characteristics extracted were publication date, journal name and impact factor; medical condition of interest; reporting of protocol availability (i.e., registration or publication); any involvement of epidemiologists or statisticians as defined elsewhere^49^; funding source and declared conflicts of interest by the review authors; and main objective of the study, expressed as population, intervention or exposure, control group and outcome (PICO^18^), explicitly provided by the authors or extracted from the text of the article. We also evaluated whether a primary outcome was defined.

Data extracted for the systematic review process were first those related to the search strategy: interrogated electronic databases, reporting of the search algorithm for at least one database, and eventual use of an animal filter^47,50^; whether restriction of language or publication date was applied, search of reference lists of articles or relevant reviews, manual search of conference abstracts, books or specific journals, investigation of the “grey” literature (Google search engine queries, dedicated websites, and unpublished data obtained by contacting experts in the field). We then assessed the selection and data extraction process: whether eligibility criteria were defined and whether these steps were performed in duplicate; the evaluation of the methodological quality of included studies and which tool was used^22,51,52^; number of screened, included and excluded studies and reasons for study exclusion reported in a flow chart; and number of animal and human studies.

Meta-analyses data extracted were the effect size measure; statistical model used to pool data (fixed-effects and/or random-effects model), and whether it was chosen according to a heterogeneity statistic; evaluation of heterogeneity; exploration of heterogeneity and if so, by which means (subgroup analysis, meta-regression model); assessment of the potential impact of the methodological quality of included studies on the meta-analysis result (subgroup analysis according to the methodological quality, meta-regression, sensitivity analyses excluding low quality studies); and investigation of the small-study effects by a funnel plot, by tests for funnel plot asymmetry, or by using the trim-and-fill method^53^ if the number of studies was sufficient. For characteristics that may vary across meta-analyses (e.g., effect size measure), we focused on the primary outcome or the first reported if no primary outcome was defined.

#### Characteristics of included preclinical studies

We focused on systematic reviews and meta-analyses reporting an evaluation of the methodological quality for each included study and a standardized mean difference (SMD) as the effect size for the primary or first reported outcome (this sample was also used for the subsequent meta-epidemiological analysis). For each included study, we collected the language of publication, the country of the laboratory (based on the affiliation of the last author) and the species used for experimentation. We extracted features related to the risk of bias, adapted from the SYRCLE risk of bias tool for animal studies^22^: reporting of randomization, and if available, appropriate sequence generation; similar group characteristics at baseline; appropriate allocation concealment; random housing of animals; blinding of caregivers; blinding of the animal model; random assessment of outcomes; blinding of assessors; and adequate management of incomplete outcome data and presence of selective outcome reporting. The quality of reporting and methods of experimental procedures were rated according to the CAMARADES quality items^51^: publication in a peer-reviewed journal, reporting of control of temperature during experimental procedure, use of an anesthetic without protective effect on outcome, description of the animal model, sample size calculation, compliance with animal welfare regulations and statement of conflicts of interest.

The value of each individual study SMD and its confidence interval for the primary or first reported outcome was also collected for the meta-epidemiological analysis.

All data were extracted directly from the reviews, except language of publication and country of laboratory, which were retrieved from the original publication.

### Statistical analysis

#### Descriptive analysis

Categorical variables were expressed as number (percentage) and quantitative variables as median (Q1, Q3). Characteristics of meta-analyses evaluating a therapeutic intervention and those exploring the pathophysiology of a disease were described separately.

#### Meta-epidemiological analysis

The association between the effect size and each key methodological characteristic related to risk of bias as defined above for included studies was explored by a two-step meta-epidemiological approach^54^ (for an overview of the statistical methodology, see Supplementary Fig. S1). First, for each meta-analysis, we used random-effects meta-regression to estimate the difference in the reported effect size (i.e., SMD) between studies at high or unclear risk for the methodological characteristic and the SMD of studies at low risk (difference in standardized mean difference [DMSD]). To obtain comparable effect sizes, all SMDs were transformed before meta-regression to be positive in case of a beneficial treatment effect (or expected direction of the relation for studies exploring the pathophysiology of a disease). Second, the DMSDs obtained per meta-analysis were combined by a random-effects meta-analysis^55^. This combined estimate provides an estimation of the average association between the methodological characteristic and the effect size across meta-analyses. A positive combined estimate of the DMSD suggests a larger effect size for studies at high or unclear risk of bias for the characteristic. In contrast, a negative combined DMSD indicates that a smaller effect size is observed in studies with poor methodological features.

We performed the meta-epidemiological analysis if there were at least 10 eligible meta-analyses for a given characteristic. An eligible meta-analysis was a meta-analysis including ≥ 3 studies with at least one study at high or unclear risk of bias and one study at low risk of bias for the studied characteristic.

Because numerous meta-analyses included several times the same study as multiple experimental arms, we performed a sensitivity analysis for estimating the DMSD by using a meta-regression with a robust variance estimator, which takes into account the dependency of the different experimental arms of the same study included in the meta-analysis^15,56^.

Statistical analysis was performed with R 3.6 (R Core Team (2019). R: A language and environment for statistical computing. R Foundation for Statistical Computing, Vienna, Austria. URL https://www.R-project.org/).

## Supplementary Information

Supplementary Information.

## Data Availability

The datasets generated and analyzed during the current study are not publicly available due to current unpublished works with them, but are available from the corresponding author on reasonable request.
