# Supplementary material for: A methodological review with meta-epidemiological analysis of preclinical systematic reviews with meta-analyses
Source: Sci Rep. 2022 Nov 21;12:20066. doi: 10.1038/s41598-022-24447-4 (PMC9681751; doi:10.1038/s41598-022-24447-4)
Supplement: Supplementary file 1 — Supplementary Information. [file 41598_2022_24447_MOESM1_ESM.docx]

**Supporting Information**

**Supplementary File S1.** **Search equation for identification of studies in MEDLINE via PubMed.**

**Supplementary File S2. List of included systematic reviews with meta-analyses in the methodological review.**

**Supplementary file S3. Supplemental references.**

**Supplementary Table S1. Definition of population, intervention or exposure, control and outcome in the included systematic reviews.**

**Supplementary Table S2.** **Characteristics of the systematic reviews with meta-analyses including both animal and human studies.**

**Supplementary Table S3.** **Summary of results of the systematic reviews with meta-analyses.**

**Supplementary Table S4. Summary of results of meta-epidemiological analysis.**

**Supplementary Figure S1. Two-step meta-epidemiological analysis [1]**. SMD: standardized mean difference; DSMD: difference in standardized mean difference.

**Supplementary Figure S2.** **Selection process of studies included in the methodological review of included studies and the meta-epidemiological analysis**. Adapted from PRISMA flow diagram [2]. SMD: standardized mean difference.

**Supplementary Figure S3. Difference in standardized mean difference (SMD) for risk of bias estimated by RVE meta-regression.** A positive difference in SMD reveals a larger effect size in studies at high or unclear risk of bias. A negative difference in SMD indicates a smaller effect size in case of threats to methodological quality. Het: heterogeneity; MA: meta-analyses; RVE: robust variance estimator.

**Supplementary File S1.** **Search equation for identification of studies in Medline via PubMed.**

("Meta-analysis "[title] OR “Meta-analyses”[title] OR "Metaanalysis"[title] OR " Metaanalyses"[title] OR " Meta-analysis"[pt] OR ("Meta-analysis"[tiab] OR " Meta-analyses"[tiab] OR "Metaanalysis"[tiab] OR " Metaanalyses"[tiab] NOT medline[subset]))

AND

(preclinical[tiab] OR “in vivo”[tiab] OR “animal”[tiab] OR animals[tiab] OR mice[tiab] OR mus[tiab] OR mouse[tiab] OR murine[tiab] OR woodmouse[tiab] OR rats[tiab] OR rat[tiab] OR murinae[tiab] OR muridae[tiab] OR cottonrat[tiab] OR cottonrats[tiab] OR hamster[tiab] OR hamsters[tiab] OR cricetinae[tiab] OR "guinea pigs"[tiab] OR "guinea pig"[tiab]OR rodentia[tiab] OR rodent[tiab] OR rodents[tiab] OR eel[tiab] OR eels[tiab] OR fish[tiab] OR fishes[tiab] OR pisces[tiab] OR catfish[tiab] OR catfishes[tiab] OR siluriformes[tiab] OR arius[tiab] OR heteropneustes[tiab] OR sheatfish[tiab] OR perch[tiab] OR perches[tiab] OR percidae[tiab] OR perca[tiab] OR trout[tiab] OR trouts[tiab] OR char[tiab] OR chars[tiab] OR salvelinus[tiab] OR "fathead minnow"[tiab] OR minnow[tiab] OR cyprinidae[tiab] OR carps[tiab] OR carp[tiab] OR zebrafish[tiab] OR zebrafishes[tiab] OR goldfish[tiab] OR goldfishes[tiab] OR guppy[tiab] OR guppies[tiab] OR chub[tiab] OR chubs[tiab] OR tinca[tiab] OR barbels[tiab] OR barbus[tiab] OR pimephales[tiab] OR promelas[tiab] OR "poecilia reticulata"[tiab] OR mullet[tiab] OR mullets[tiab] OR seahorse[tiab] OR seahorses[tiab] OR mugil curema[tiab] OR atlantic cod[tiab] OR shark[tiab] OR sharks[tiab] OR catshark[tiab] OR anguilla[tiab] OR salmonid[tiab] OR salmonids[tiab] OR whitefish[tiab] OR whitefishes[tiab] OR salmon[tiab] OR salmons[tiab] OR sole[tiab] OR solea[tiab] OR "sea lamprey"[tiab] OR lamprey[tiab] OR lampreys[tiab] OR pumpkinseed[tiab] OR sunfish[tiab] OR sunfishes[tiab] OR tilapia[tiab] OR tilapias[tiab] OR turbot[tiab] OR turbots[tiab] OR flatfish[tiab] OR flatfishes[tiab])

NOT

(“protocol”[title] OR “case reports”[pt] OR “Comment”[pt] OR “Editorial”[pt] OR Case-Control Studies[mh] OR "cohort studies"[title] OR longitudinal[title] OR diagnostic[title]  OR biomarkers[title] OR biomarker[title] OR veterinary[title] OR “Veterinary Medicine”[mh] OR veterinarian[tiab] OR cattle[tiab] OR dairy-cow[tiab] OR “dairy-cows”[tiab] OR “dairy cow”[tiab] OR “dairy cows”[tiab] OR "Diet/veterinary"[mh] OR "Dairying"[mh] OR “Horse Diseases”[mh] OR “Cattle Diseases”[mh] OR “Cat Diseases”[mh] OR “Dog Diseases”[mh] OR “Fish Diseases”[mh] OR “Sheep Diseases”[mh] OR “Swine Diseases”[mh] OR Zoonoses[mh] OR ecological[title] OR ecology[title] OR anthropogenic[tiab] OR agriculture[tiab] OR agricultural[tiab] OR soil[tiab] OR soils[tiab] OR geographically[tiab] OR geographical[tiab] OR Geography[mh] OR “Water pollutants”[mh] OR herbivore[tiab] OR carnivore[tiab] OR “Animals, Wild*”[mh] OR wildlife[tiab] OR “Predatory Behavior”[mh] OR ecosystem[title] OR biodiversity[tiab] OR pollination[tiab] OR “Insect Control”[mh] OR “Disease Vectors”[mh]OR "Climate Change"[mh] OR “Food Handling”[mh] OR “Dairy Product”[tiab] OR “Dairy Products”[tiab] OR “Food Contamination”[mh] OR SNP[tiab] OR SNPs[tiab] OR "Genome-wide"[tiab] OR “Phenome-wide”[tiab] OR “genetic variation”[tiab] OR “genetic testing”[tiab] OR seroprevalence[tiab] OR “Seroepidemiologic Studies”[MesH] OR “transcriptional profiling”[tiab] OR transcriptome[tiab] OR RNA-seq[tiab] OR “Gene Expression Profiling”[mh] OR microarray[tiab] OR microarrays[tiab] OR “gene set enrichment”[tiab] OR (genetic[tiab] AND polymorphism[tiab]) OR “Gene Expression”[mh] OR “Precision Medicine*”[mh] OR Genomics[mh]  OR transcriptomic[tiab])

**Note**. We chose to design our search equation with the synonyms of the terms mice, rats and fishes as these animals are the most used in the setting of preclinical research ^3^. These terms derived from the animal filter previously published for PubMed ^4^.

**Supplementary File S2. List of included systematic reviews with meta-analyses in the methodological review.** PMID: PubMed Identifier.

| **PMID** | **Title** | **First Author** |
| --- | --- | --- |
| 32020483 | Checkpoint inhibitor therapy in preclinical sepsis models: a systematic review and meta-analysis. | Busch LM |
| 32019582 | Efficacy of mesenchymal stem cells in animal models of lupus nephritis: a meta-analysis. | Zhou T |
| 32009890 | Kaixinsan, a Well-Known Chinese Herbal Prescription, for Alzheimer's Disease and Depression: A Preclinical Systematic Review. | Fu H |
| 32006552 | Revisiting the validity of the mouse tail suspension test: Systematic review and meta-analysis of the effects of prototypic antidepressants. | Stukalin Y |
| 31998433 | Efficacy of Curcumin on Aortic Atherosclerosis: A Systematic Review and Meta-Analysis in Mouse Studies and Insights into Possible Mechanisms. | Lin K |
| 31992124 | The effect of dietary protein intake on factors associated with male infertility: A systematic literature review and meta-analysis of animal clinical trials in rats. | Ajuogu PK |
| 31991178 | Exercise effects on brain and behavior in healthy mice, Alzheimer's disease and Parkinson's disease model-A systematic review and meta-analysis. | da Costa Daniele TM |
| 31987779 | Praziquantel versus praziquantel associated with immunomodulators in mice infected with schistosoma mansoni: A systematic review and meta-analy^6^sis. | Silva JCS |
| 31983304 | Conflicting Effects of Fetal Growth Restriction on Blood Pressure Between Human and Rat Offspring: A Meta-Analysis. | Kooiman J |
| 31978520 | Protective effect and possible mechanisms of ligustrazine isolated from Ligusticum wallichii on nephropathy in rats with diabetes: A preclinical systematic review and meta-analysis. | Zhuang Z |
| 31977931 | A systematic review and meta-analysis of pregabalin preclinical studies. | Federico CA |
| 31967282 | Prior exercise training and experimental myocardial infarction: A systematic review and meta-analysis. | Veiga ECA |
| 31953652 | Meta-analysis of targeted temperature management in animal models of cardiac arrest. | Olai H |
| 31939049 | Physical exercise effects on metastasis: a systematic review and meta-analysis in animal cancer models. | Rincón-Castanedo C |
| 31934329 | Does a high-fat diet affect the development and progression of osteoarthritis in mice?: A systematic review. | Sansone V |
| 31920660 | A Significant Association Between Rhein and Diabetic Nephropathy in Animals: A Systematic Review and Meta-Analysis. | Hu HC |
| 31915451 | Therapeutic Effect of Tanshinone IIA on Liver Fibrosis and the Possible Mechanism: A Preclinical Meta-Analysis. | Ying Q |
| 31900218 | Extracellular vesicles for acute kidney injury in preclinical rodent models: a meta-analysis. | Liu C |
| 31890250 | The capacity for oestrogen to influence obesity through brown adipose tissue thermogenesis in animal models: A systematic review and meta-analysis. | Sievers W |
| 31884321 | Prenatal stress and later metabolic consequences: Systematic review and meta-analysis in rodents. | Burgueño AL |
| 31882078 | The antidiabetic effect of thymoquinone: A systematic review and meta-analysis of animal studies. | Bule M |
| 31855708 | Pharmacological interventions to reduce edema following cardiopulmonary bypass: A systematic review and meta-analysis. | Dekker NAM |
| 31849654 | Astragaloside IV Exerts Cardioprotection in Animal Models of Viral Myocarditis: A Preclinical Systematic Review and Meta-Analysis. | Zhuang Z |
| 31848960 | Impact of chlorpyrifos on blood glucose concentration in an animal model: a systematic review and meta-analysis. | Farkhondeh T |
| 31845042 | Laser photobiomodulation for cartilage defect in animal models of knee osteoarthritis: a systematic review and meta-analysis. | Xiang A |
| 31844940 | Effect of Platelet-Rich Fibrin on Fat Grafting in Animal Models: A Meta-Analysis. | Liu R |
| 31841831 | Lead-induced oxidative damage in rats/mice: A meta-analysis. | Fan Y |
| 31837360 | Neuropathic pain after spinal cord injury and physical exercise in animal models: A systematic review and meta-analysis. | Palandi J |
| 31823893 | Therapeutic effect of regulating autophagy in spinal cord injury: a network meta-analysis of direct and indirect comparisons. | Zhang D |
| 31800057 | Effect of vitamins C and E on recovery of motor function after spinal cord injury: systematic review and meta-analysis of animal studies. | Hosseini M |
| 31796777 | Adhesion reformation and the limited translational value of experiments with adhesion barriers: A systematic review and meta-analysis of animal models. | Strik C |
| 31788795 | Regulatory T cells for amyotrophic lateral sclerosis/motor neuron disease: A clinical and preclinical systematic review. | Rajabinejad M |
| 31788761 | Statin Therapy in Ischemic Stroke Models: A Meta-Analysis. | Christophe B |
| 31785874 | Effect of dietary probiotics on colon length in an inflammatory bowel disease-induced murine model: A meta-analysis. | Ahn SI |
| 31773996 | Navigating to the most promising directions amid complex fields of vaccine development: a chlamydial case study. | Lizárraga D |
| 31772391 | The effect of analgesics on stimulus evoked pain-like behaviour in animal models for chemotherapy induced peripheral neuropathy- a meta-analysis. | Hooijmans CR |
| 31747888 | Bone mesenchymal stem cell therapy for ovariectomized osteoporotic rats: a systematic review and meta-analysis. | Jin Z |
| 31733955 | Effects of short-term exposure to particulate matters on heart rate variability: A systematic review and meta-analysis based on controlled animal studies. | Huang F |
| 31681006 | Ginkgolide B for Myocardial Ischemia/Reperfusion Injury: A Preclinical Systematic Review and Meta-Analysis. | Zhu PC |
| 31678635 | Therapeutic efficacy of mesenchymal stromal cells and secretome in pulmonary arterial hypertension: A systematic review and meta-analysis. | Muhammad SA |
| 31675724 | A combination of mesenchymal stem cells and scaffolds promotes motor functional recovery in spinal cord injury: a systematic review and meta-analysis. | Yousefifard M |
| 31673885 | Therapies to limit myocardial injury in animal models of myocarditis: a systematic review and meta-analysis. | Silverblatt JA |
| 31666086 | Mesenchymal stromal cell conditioned media for lung disease: a systematic review and meta-analysis of preclinical studies. | Emukah C |
| 31663000 | Effects of Systemic or Local Administration of Zoledronate on Implant Osseointegration: A Preclinical Meta-Analysis. | He Y |
| 31659548 | Comparison of coated meshes for intraperitoneal placement in animal studies: a systematic review and meta-analysis. | Liu H |
| 31654281 | From the Lab to Patients: a Systematic Review and Meta-Analysis of Mesenchymal Stem Cell Therapy for Stroke. | Lalu MM |
| 31647992 | Metabolic and behavioural effects of prenatal exposure to non-nutritive sweeteners: A systematic review and meta-analysis of rodent models. | Morahan HL |
| 31638100 | Effects of neural stem cell transplantation on the motor function of rats with contusion spinal cord injuries: a meta-analysis. | Qian K |
| 31605011 | Macronutrient Supplements in Preterm and Small-for-Gestational-Age Animals: A Systematic Review and Meta-analysis. | Amissah E |
| 31600860 | Doxorubicin-induced skeletal muscle atrophy: Elucidating the underlying molecular pathways. | Hiensch AE |
| 31595835 | Topical Host-Modulating Therapy for Periodontal Regeneration: A Systematic Review and Meta-Analysis. | Wang B |
| 31595204 | Preclinical Evidence and Possible Mechanisms of Extracts or Compounds from Cistanches for Alzheimer's Disease. | Zhou XL |
| 31593923 | The effect of electrical stimulation therapies on spinal fusion: a cross-disciplinary systematic review and meta-analysis of the preclinical and clinical data. | Cottrill E |
| 31581897 | The effect of angiopoietin-1 upregulation on the outcome of acute ischaemic stroke in rodent models: A meta-analysis. | Moxon JV |
| 31571666 | Effect of docosahexaenoic acid on the recovery of motor function in rats with spinal cord injury: a meta-analysis. | Tian ZR |
| 31571660 | Neurological recovery and antioxidant effects of resveratrol in rats with spinal cord injury: a meta-analysis. | Xu BP |
| 31568315 | A Systematic Review and Meta-Analysis of the Pressure-Induced Vasodilation Phenomenon and Its Role in the Pathophysiology of Ulcers. | Zwanenburg PR |
| 31527399 | The Application of Statins in the Regeneration of Bone Defects. Systematic Review and Meta-Analysis. | Roca-Millan E |
| 31507524 | The Potential of Biomaterial-Based Approaches as Therapies for Ischemic Stroke: A Systematic Review and Meta-Analysis of Pre-clinical Studies. | Bolan F |
| 31501076 | Meta-analysis of preclinical studies of mesenchymal stromal cells to treat rheumatoid arthritis. | Liu L |
| 31498296 | Glucocorticoid for Hearing Preservation After Cochlear Implantation: A Systemic Review and Meta-analysis of Animal Studies. | Shaul C |
| 31480966 | Central Nervous System Electrical Stimulation for Neuroprotection in Acute Cerebral Ischemia: Meta-Analysis of Preclinical Studies. | Bahr Hosseini M |
| 31474857 | Testing the Translational Power of the Zebrafish: An Interspecies Analysis of Responses to Cardiovascular Drugs. | Margiotta-Casaluci L |
| 31466409 | Synthetic Blocks for Bone Regeneration: A Systematic Review and Meta-Analysis. | Tumedei M |
| 31454627 | The effect of high fat, high sugar, and combined high fat-high sugar diets on spatial learning and memory in rodents: A meta-analysis. | Abbott KN |
| 31445128 | Guizhi Fuling Wan for uterine fibroids: A systematic review of in vivo studies. | Li M |
| 31443677 | Mesenchymal stem cells for the treatment of ulcerative colitis: a systematic review and meta-analysis of experimental and clinical studies. | Shi X |
| 31411119 | Bone Regeneration Using Antiosteoporotic Drugs in Adjunction with Bone Grafting: A Meta-Analysis. | Shaheen MY |
| 31392210 | Efficacy of Terpenoid in Attenuating Aortic Atherosclerosis in Apolipoprotein-E Deficient Mice: A Meta-Analysis of Animal Studies. | Liu H |
| 31391859 | Effectiveness of Acupuncture in Treatment of Simple Obesity in Animal Models: A Systematic Review and Meta-Analysis. | Fan XL |
| 31373407 | Medication-related osteonecrosis of the jaw-like lesions in rodents: A comprehensive systematic review and meta-analysis. | Kuroshima S |
| 31367162 | Bioartificial liver support systems for acute liver failure: A systematic review and meta-analysis of the clinical and preclinical literature. | He YT |
| 31342340 | Oxidative Damage Induced by Nano-titanium Dioxide in Rats and Mice: a Systematic Review and Meta-analysis. | An H |
| 31320210 | Psychotropic and neurological medication effects on mitochondrial complex I and IV in rodent models. | Holper L |
| 31290450 | MK-801 attenuates lesion expansion following acute brain injury in rats: a meta-analysis. | Yi NX |
| 31258512 | Meta-Analysis of the Effect of Exercise on Neuropathic Pain Induced by Peripheral Nerve Injury in Rat Models. | Guo JB |
| 31242880 | Microarchitectural changes in the mandibles of ovariectomized rats: a systematic review and meta-analysis. | Lee JH |
| 31232460 | Stem/progenitor cell-mediated pulpal tissue regeneration: a systematic review and meta-analysis. | Fawzy El-Sayed KM |
| 31208348 | Anticancer effect of berberine based on experimental animal models of various cancers: a systematic review and meta-analysis. | Xu J |
| 31182995 | A Meta-Analysis of Resveratrol Protects against Myocardial Ischemia/Reperfusion Injury: Evidence from Small Animal Studies and Insight into Molecular Mechanisms. | Mao ZJ |
| 31165913 | The effects of cannabinoid 1 receptor compounds on memory: a meta-analysis and systematic review across species. | Borgan F |
| 31165092 | Fever worsens outcomes in animal models of ischaemic stroke: A systematic review and meta-analysis. | de Jonge JC |
| 31163203 | The efficacy and underlying mechanism of phosphodiesterase- 5 inhibitors in preventing cognitive impairment and Alzheimer pathology: A systematic review of animal studies. | El-Bakly W |
| 31139074 | Does Acupuncture Protect Dopamine Neurons in Parkinson's Disease Rodent Model?: A Systematic Review and Meta-Analysis. | Ko JH |
| 31068894 | World-Wide Efficacy of Bone Marrow Derived Mesenchymal Stromal Cells in Preclinical Ischemic Stroke Models: Systematic Review and Meta-Analysis. | Satani N |
| 31065943 | Effect of resveratrol on metabolic syndrome components: A systematic review and meta-analysis. | Asgary S |
| 31047892 | The behavioral phenotype of early life adversity: A 3-level meta-analysis of rodent studies. | Bonapersona V |
| 31035477 | Can Resveratrol Treatment Control the Progression of Induced Periodontal Disease? A Systematic Review and Meta-Analysis of Preclinical Studies. | Andrade EF |
| 31022999 | Adipose Tissue-Derived Stem Cell Therapy for Cavernous Nerve Injury-Induced Erectile Dysfunction in the Rat Model: A Systematic Review and Meta-Analysis Using Methodological Quality Assessment. | Park HJ |
| 30980937 | The bone regeneration capacity of 3D-printed templates in calvarial defect models: A systematic review and meta-analysis. | Hassan MN |
| 30973513 | Compressive Pressure Versus Time in Cauda Equina Syndrome: A Systematic Review and Meta-Analysis of Experimental Studies. | Pronin S |
| 30916812 | Zirconia compared to titanium dental implants in preclinical studies-A systematic review and meta-analysis. | Roehling S |
| 30903654 | Progress in the Use of Induced Pluripotent Stem Cell-Derived Neural Cells for Traumatic Spinal Cord Injuries in Animal Populations: Meta-Analysis and Review. | Ramotowski C |
| 30867709 | Efficacy of bone marrow mesenchymal stem cell transplantation in animal models of pulmonary fibrosis after exposure to bleomycin: A meta-analysis. | Zhang E |
| 30841915 | Regenerative cell therapy for pulmonary arterial hypertension in animal models: a systematic review. | Suen CM |
| 30826386 | Systematic review and meta-analysis of the behavioral effects of methylphenidate in the spontaneously hypertensive rat model of attention-deficit/hyperactivity disorder. | Leffa DT |
| 30810979 | Effect of Exercise Interventions on Kainate Induced Status Epilepticus and Associated Co-morbidities; A Systematic Review and Meta-Analysis. | Iqbal M |
| 30805305 | Impact of Physical Exercise on Growth and Progression of Cancer in Rodents-A Systematic Review and Meta-Analysis. | Eschke RK |
| 30792374 | Stem cell-derived extracellular vesicles for myocardial infarction: a meta-analysis of controlled animal studies. | Yang L |
| 30786027 | Riluzole promotes neurological function recovery and inhibits damage extension in rats following spinal cord injury: a meta-analysis and systematic review. | Zhou LY |
| 30782462 | Preclinical application of recombinant human bone morphogenetic protein 2 on bone substitutes for vertical bone augmentation: A systematic review and meta-analysis. | Teng F |
| 30760312 | Efficacy of stem cell therapy for pulmonary arterial hypertension: a systematic review and meta-analysis of preclinical studies. | Ding XF |
| 30756459 | High-fat diets reduce male reproductive success in animal models: A systematic review and meta-analysis. | Crean AJ |
| 30739594 | Meta-Analysis of the Safety and Efficacy of Stem Cell Therapies for Ischemic Stroke in Preclinical and Clinical Studies. | Ouyang Q |
| 30735748 | Antidiabetic effect of quercetin: A systematic review and meta-analysis of animal studies. | Bule M |
| 30726717 | Axonal degeneration and demyelination following traumatic spinal cord injury: A systematic review and meta-analysis. | Hassannejad Z |
| 30696832 | Remyelination promoting therapies in multiple sclerosis animal models: a systematic review and meta-analysis. | Hooijmans CR |
| 30670723 | The antidepressant impact of minocycline in rodents: A systematic review and meta-analysis. | Reis DJ |
| 30658106 | Influence of antimicrobial photodynamic therapy as an adjunctive to scaling and root planing on alveolar bone loss: A systematic review and meta-analysis of animal studies. | Alberton Nuernberg MA |
| 30633422 | Maternal obesity in pregnancy impacts offspring cardiometabolic health: Systematic review and meta-analysis of animal studies. | Menting MD |
| 30623455 | Cell therapy for orofacial bone regeneration: A systematic review and meta-analysis. | Shanbhag S |
| 30588304 | Systematic review and meta-analysis of the protective effect of resveratrol on multiple organ injury induced by sepsis in animal models. | Zhou J |
| 30529501 | Helminth-based therapies for rheumatoid arthritis: A systematic review and meta-analysis. | Langdon K |
| 30520038 | Effects of cannabinoids in Amyotrophic Lateral Sclerosis (ALS) murine models: a systematic review and meta-analysis. | Urbi B |
| 30517943 | Pericardium in Reconstructive Urologic Surgeries: A Systematic Review and Meta-Analysis. | Hosseini J |
| 30496803 | Effect of mesenchymal stromal (stem) cell (MSC) transplantation in asthmatic animal models: A systematic review and meta-analysis. | Zhang LB |
| 30489210 | The effect of rapamycin treatment on cerebral ischemia: A systematic review and meta-analysis of animal model studies. | Beard DJ |
| 30484018 | Chinese Herbal Medicine for Osteosarcoma in the Mouse: A Systematic Review and Meta-Analysis. | Chang JL |
| 30362597 | Effects of alpha lipoic acid and its derivative "andrographolid-lipoic acid-1" on ulcerative colitis: A systematic review with meta-analysis of animal studies. | Moeinian M |
| 30357758 | The Variable Regulatory Effect of Arsenic on Nrf2 Signaling Pathway in Mouse: a Systematic Review and Meta-analysis. | Wang C |
| 30326252 | Endurance training on rodent brain antioxidant capacity: A meta-analysis. | de Souza RF |
| 30317001 | The protective effect of anterior cruciate ligament reconstruction on articular cartilage: a systematic review of animal studies. | Deckers C |
| 30315826 | The relationship between oxytocin, dietary intake and feeding: A systematic review and meta-analysis of studies in mice and rats. | Skinner JA |
| 30247648 | Cell-Enhanced Acellular Nerve Allografts for Peripheral Nerve Reconstruction: A Systematic Review and a Meta-Analysis of the Literature. | Pedrini FA |
| 30191772 | Antiosteoporotic Drugs to Promote Bone Regeneration Related to Titanium Implants: A Systematic Review and Meta-Analysis. | Basudan AM |
| 30084022 | Comparative efficacy of stem cells and secretome in articular cartilage regeneration: a systematic review and meta-analysis. | Muhammad SA |
| 30062923 | Growth changes after inhalant abuse and toluene exposure: A systematic review and meta-analysis of human and animal studies. | Crossin R |
| 29901413 | Melatonin as a Treatment after Traumatic Brain Injury: A Systematic Review and Meta-Analysis of the Pre-Clinical and Clinical Literature. | Barlow KM |
| 30619794 | A Review of Oxygen Use During Chest Compressions in Newborns-A Meta-Analysis of Animal Data. | Garcia-Hidalgo C |
| 30619060 | Efficacy of Minocycline in Acute Ischemic Stroke: A Systematic Review and Meta-Analysis of Rodent and Clinical Studies. | Sheng Z |
| 30581490 | Preclinical Evidence and Mechanism of Xingnaojing Injection for Cerebral Ischemia: A Systematic Review and Meta-Analysis of Animal Studies. | Ma R |
| 30564123 | Rhodiola rosea L. Improves Learning and Memory Function: Preclinical Evidence and Possible Mechanisms. | Ma GP |
| 30562321 | Therapeutic effects of whole-body vibration on fracture healing in ovariectomized rats: a systematic review and meta-analysis. | Chen J |
| 30483348 | Intracerebral Adenosine During Sleep Deprivation: A Meta-Analysis and New Experimental Data. | Leenaars CHC |
| 30473742 | Relevance of Trypanothione Reductase Inhibitors on Trypanosoma cruzi Infection: A Systematic Review, Meta-Analysis, and In Silico Integrated Approach. | Mendonça AAS |
| 30466079 | Systematic Review and Meta-Analysis of the Impact of Hypoxia on Infarcted Myocardium: Better or Worse? | Pang B |
| 30424749 | Effects of acupuncture on vascular dementia (VD) animal models: a systematic review and meta-analysis. | Zhang ZY |
| 30369951 | Intraparenchymal Neural Stem/Progenitor Cell Transplantation for Ischemic Stroke Animals: A Meta-Analysis and Systematic Review. | Huang H |
| 30352012 | Polybrominated diphenyl ether (PBDE) neurotoxicity: a systematic review and meta-analysis of animal evidence. | Dorman DC |
| 30310710 | Stem cell transplantation and functional recovery after spinal cord injury: a systematic review and meta-analysis. | Abbaszadeh HA |
| 30283040 | Effect of statins on experimental postoperative adhesion: a systematic review and meta-analysis. | Choi GJ |
| 30255101 | Optimizing Livers for Transplantation Using Machine Perfusion versus Cold Storage in Large Animal Studies and Human Studies: A Systematic Review and Meta-Analysis. | Jiang X |
| 30201218 | Effects of early life stress on biochemical indicators of the dopaminergic system: A 3 level meta-analysis of rodent studies. | Bonapersona V |
| 30199890 | Meta-analysis on the effect of hyperbaric oxygen as adjunctive therapy in the outcome of anastomotic healing of experimental colorectal resections in rats. | Brouwer RJ |
| 30199328 | Systematic reviews and meta-analyses of human and animal evidence of prenatal diethylhexyl phthalate exposure and changes in male anogenital distance. | Dorman DC |
| 30177934 | Meta-Analysis of Preclinical Studies of Fibrinolytic Therapy for Acute Lung Injury. | Liu C |
| 30159323 | Programmed Cell Death-1/Programmed Death-Ligand 1 Blockade Improves Survival of Animals with Sepsis: A Systematic Review and Meta-Analysis. | Zhang Q |
| 30145225 | Systematic review and meta-analysis of interventions tested in animal models of pulmonary hypertension. | Sztuka K |
| 30116515 | Use of Autologous Stem Cells for the Regeneration of Periodontal Defects in Animal Studies: a Systematic Review and Meta-Analysis. | Gaubys A |
| 30106427 | The role of C-peptide in the attenuation of outcomes of diabetic kidney disease: a systematic review and meta-analysis. | Oliveira CM |
| 30090062 | Ligustrazine Exerts Cardioprotection in Animal Models of Myocardial Ischemia/Reperfusion Injury: Preclinical Evidence and Possible Mechanisms. | Zheng Q |
| 30075723 | Effects of hyperoxia on vascular tone in animal models: systematic review and meta-analysis. | Smit B |
| 30053633 | Systematic review and meta-analysis of preclinical trials demonstrate robust beneficial effects of prebiotics in induced inflammatory bowel disease. | Rufino MN |
| 30018562 | A Preclinical Systematic Review and Meta-Analysis of Astragaloside IV for Myocardial Ischemia/Reperfusion Injury. | Zheng Q |
| 29961052 | Induced Pluripotent Stem Cell Transplantation Improves Locomotor Recovery in Rat Models of Spinal Cord Injury: a Systematic Review and Meta-Analysis of Randomized Controlled Trials. | Qin C |
| 29957455 | The impact of dietary consistency on structural craniofacial components: Temporomandibular joint/condyle, condylar cartilage, alveolar bone and periodontal ligament. A systematic review and meta-analysis in experimental in vivo research. | Scheidegger R |
| 29926838 | Myelotomy promotes locomotor recovery in rats subjected to spinal cord injury: A meta-analysis of six randomized controlled trials. | Qin C |
| 29924822 | The anxiolytic effect of probiotics: A systematic review and meta-analysis of the clinical and preclinical literature. | Reis DJ |
| 29922450 | Tissue engineering strategies for the treatment of tendon injuries: a systematic review and meta-analysis of animal models. | González-Quevedo D |
| 29922166 | Isoquinoline Alkaloids and Indole Alkaloids Attenuate Aortic Atherosclerosis in Apolipoprotein E Deficient Mice: A Systematic Review and Meta-Analysis. | Zhang Y |
| 29908308 | Efficacy of whole-cell pneumococcal vaccine in mice: A systematic review and meta-analysis. | Mohammadzadeh M |
| 29877067 | Effects of mesenchymal stem cells transplantation on cognitive deficits in animal models of Alzheimer's disease: A systematic review and meta-analysis. | Ge M |
| 29861768 | Effects of Panax ginseng on Obesity in Animal Models: A Systematic Review and Meta-Analysis. | Park HS |
| 29849531 | Normothermic Ex Vivo Machine Perfusion for Liver Grafts Recovered from Donors after Circulatory Death: A Systematic Review and Meta-Analysis. | Nostedt JJ |
| 29772024 | Use of human amniotic epithelial cells in mouse models of bleomycin-induced lung fibrosis: A systematic review and meta-analysis. | He F |
| 29759026 | Efficacy of Oligodendrocyte Progenitor Cell Transplantation in Rat Models with Traumatic Thoracic Spinal Cord Injury: A Systematic Review and Meta-Analysis. | Fu H |
| 29755593 | Effect of stem cell transplantation of premature ovarian failure in animal models and patients: A meta-analysis and case report. | Chen L |
| 29703232 | Effects of mesenchymal stem cells on solid tumor metastasis in experimental cancer models: a systematic review and meta-analysis. | Li JH |
| 29643818 | Intrinsic Hormone-Like Molecules and External Root Resorption During Orthodontic Tooth Movement. A Systematic Review and Meta-Analysis in Preclinical in-Vivo Research. | Spoerri A |
| 29642387 | A Beneficial Role of Rooibos in Diabetes Mellitus: A Systematic Review and Meta-Analysis. | Sasaki M |
| 29617793 | The effect of biologic factors and adjunctive therapies on orthodontically induced inflammatory root resorption: a systematic review and meta-analysis. | Haugland L |
| 29603108 | Should open excisions and sutured incisions be treated differently? A review and meta-analysis of animal wound models following low-level laser therapy. | Gál P |
| 29566055 | Mesenchymal stem cell therapy for paraquat poisoning: A systematic review and meta-analysis of preclinical studies. | He F |
| 29564842 | Efficacy of Exercise on Breast Cancer Outcomes: A Systematic Review and Meta-analysis of Preclinical Data. | Figueira ACC |
| 29564126 | Induced pluripotent stem cells in rat models of Parkinson's disease: A systematic review and meta-analysis. | Zhang Y |
| 29562280 | The Use of Cannabinoids in Colitis: A Systematic Review and Meta-Analysis. | Couch DG |
| 29541421 | A systematic review and meta-analysis of topoisomerase inhibition in pre-clinical glioma models. | Jue TR |
| 29535565 | The Efficacy and Underlying Mechanism of Moxibustion in Preventing Cognitive Impairment: A Systematic Review of Animal Studies. | Choe S |
| 29525285 | Prenatal exposure to bisphenol A and hyperactivity in children: a systematic review and meta-analysis. | Rochester JR |
| 29480934 | A Systematic Review and Quantitative Meta-Analysis of Oxytocin's Effects on Feeding. | Leslie M |
| 29457740 | Comparison of the Use of Titanium-Zirconium Alloy and Titanium Alloy in Dental Implants: A Systematic Review and Meta-Analysis. | Cruz RS |
| 29453349 | Ultrasound and shock-wave stimulation to promote axonal regeneration following nerve surgery: a systematic review and meta-analysis of preclinical studies. | Daeschler SC |
| 29436856 | Chondroitinase ABC for neurological recovery after acute brain injury: systematic review and meta-analyses of preclinical studies. | Koh CH |
| 29396596 | Subcutaneous injection of multipotent mesenchymal stromal cells admixed with melanoma cells in mice favors tumor incidence and growth: a systematic review and meta-analysis. | Cordeiro MF |
| 29385080 | A Systematic Review and Meta-Analysis of the In Vivo Haemodynamic Effects of Δ⁸-Tetrahydrocannabinol. | Sultan SR |
| 29362430 | Efficacy of bisphosphonates in the treatment of femoral head osteonecrosis: A PRISMA-compliant meta-analysis of animal studies and clinical trials. | Li D |
| 29340346 | Effects of Local Drug and Chemical Compound Delivery on Bone Regeneration Around Dental Implants in Animal Models: A Systematic Review and Meta-Analysis. | Alenezi A |
| 29334694 | Home alone: a systematic review and meta-analysis on the effects of individual housing on body weight, food intake and visceral fat mass in rodents. | Schipper L |
| 29324848 | Platelet-rich plasma to treat experimentally-induced skin wounds in animals: A systematic review and meta-analysis. | Tambella AM |
| 29321494 | Transplantation of olfactory ensheathing cells on functional recovery and neuropathic pain after spinal cord injury; systematic review and meta-analysis. | Nakhjavan-Shahraki B |
| 29313133 | Influence of subcrestal implant placement compared with equicrestal position on the peri-implant hard and soft tissues around platform-switched implants: a systematic review and meta-analysis. | Valles C |
| 29242986 | Pre- and postconditioning the heart with hydrogen sulfide (H(2)S) against ischemia/reperfusion injury in vivo: a systematic review and meta-analysis. | Karwi QG |
| 29235025 | Effects of exposure to malathion on blood glucose concentration: a meta-analysis. | Ramirez-Vargas MA |
| 29233582 | Influence of involuntary cigarette smoke inhalation on osseointegration: a systematic review and meta-analysis of preclinical studies. | Javed F |
| 29211309 | Statins in nonsurgical and surgical periodontal therapy. A systematic review and meta-analysis of preclinical in vivo trials. | Bertl K |
| 29096932 | Does incorporating collagen and chondroitin sulfate matrix in implant surfaces enhance osseointegration? A systematic review and meta-analysis. | Kellesarian SV |
| 29090465 | Mesenchymal Stem Cell Therapy in Ischemic Stroke: A Meta-analysis of Preclinical Studies. | Sarmah D |
| 28987515 | Does exercise augment operant and Pavlovian extinction: A meta-analysis. | Roquet RF |
| 28972576 | The effects of ketamine on dopaminergic function: meta-analysis and review of the implications for neuropsychiatric disorders. | Kokkinou M |
| 28949953 | Effects of probiotics on experimental necrotizing enterocolitis: a systematic review and meta-analysis. | Athalye-Jape G |
| 28760085 | The impact of RAGE inhibition in animal models of bacterial sepsis: a systematic review and meta-analysis. | Zhao X |
| 28355084 | Effect of NSAIDs on Recovery From Acute Skeletal Muscle Injury: A Systematic Review and Meta-analysis. | Morelli KM |
| 28095650 | Bone tissue engineering in oral peri-implant defects in preclinical in vivo research: A systematic review and meta-analysis. | Shanbhag S |
| 31657265 | Metabolic Remodeling in the Pressure-Loaded Right Ventricle: Shifts in Glucose and Fatty Acid Metabolism-A Systematic Review and Meta-Analysis. | Koop AC |
| 31033823 | Unconventional Perfusion Flaps in the Experimental Setting: A Systematic Review and Meta-Analysis. | Casal D |
| 30412818 | The role of phospholipase A2 in multiple Sclerosis: A systematic review and meta-analysis. | Trotter A |
| 29158125 | Sex differences in nicotine intravenous self-administration: A meta-analytic review. | Flores RJ |
| 29520012 | Assisted delivery of antisense therapeutics in animal models of heritable neurodegenerative and neuromuscular disorders: a systematic review and meta-analysis. | van der Bent ML |
| 29128579 | Revisiting the validity of the mouse forced swim test: Systematic review and meta-analysis of the effects of prototypic antidepressants. | Kara NZ |
| 31747552 | Pharmacological manipulations of judgement bias: A systematic review and meta-analysis. | Neville V |
| 31578930 | Therapeutic Approaches for Volumetric Muscle Loss Injury: A Systematic Review and Meta-Analysis. | Greising SM |
| 29416552 | Fructo-oligosaccharides and glucose homeostasis: a systematic review and meta-analysis in animal models. | Le Bourgot C |

**Supplementary File S3. Supplemental references.**

1. Sterne, J. A. C. *et al.* Statistical methods for assessing the influence of study characteristics on treatment effects in ‘meta-epidemiological’ research. *Stat. Med.* 21, 1513–1524 (2002).

2. Moher, D., Liberati, A., Tetzlaff, J., Altman, D. G., & PRISMA Group. Preferred reporting items for systematic reviews and meta-analyses: the PRISMA statement. *PLoS Med.* 6, e1000097 (2009).

3. *Annual Statistics of Scientific Procedures on Living Animals, Great Britain 2018*. (2019).

4. Hooijmans, C. R., Tillema, A., Leenaars, M. & Ritskes-Hoitinga, M. Enhancing search efficiency by means of a search filter for finding all studies on animal experimentation in PubMed. *Lab. Anim.* 44, 170–175 (2010).

**Supplementary Table S1. Definition of population, intervention or exposure, control and outcome in the included systematic reviews.**

|  | **Overall** | **Therapeutic intervention** | **Pathophysiology** |
| --- | --- | --- | --- |
|  | **(n=212)** | **(n=165)** | **(n=47)** |
| **Population** |  |  |  |
| Defined | 206 (97.2%) | 159 (96.4%) | 47 (100%) |
| Not defined | 0 (0%) | 0 (0%) | 0 (0%) |
| Unclear | 6 (2.8%) | 6 (3.6%) | 0 (0%) |
| **Intervention/exposure** |  |  |  |
| Defined | 207 (97.6%) | 162 (98.2%) | 45 (95.7%) |
| Not defined | 0 (0%) | 0 (0%) | 0 (0%) |
| Unclear | 5 (2.4%) | 3 (1.8%) | 2 (4.3%) |
| **Control** |  |  |  |
| Defined | 125 (59.0%) | 101 (61.2%) | 24 (51.1%) |
| Not defined | 60 (28.3%) | 45 (27.3%) | 15 (31.9%) |
| Unclear | 27 (12.7%) | 19 (11.5%) | 8 (17.0%) |
| **Outcome** |  |  |  |
| Defined | 72 (34.0%) | 56 (33.9%) | 16 (34.0%) |
| Not defined | 2 (0.9%) | 1 (0.6%) | 1 (2.1%) |
| Unclear | 138 (65.1%) | 108 (65.5%) | 30 (63.8%) |
| **Outcomes presented in a hierarchical way** | 110 (51.9%) | 93 (56.4%) | 17 (36.2%) |
| **Number of outcomes, median [Q1, Q3]** | 3.00 [1.75, 5.25] | 3.00 [1.00, 5.00] | 4.00 [2.00, 9.00] |
| **Timepoint of measure of outcome defined** | 72 (34.0%) | 55 (33.3%) | 17 (36.2%) |

**Supplementary Table S2.** **Characteristics of the systematic reviews with meta-analyses including both animal and human studies.**

|  | **Overall** | **Therapeutic intervention** | **Pathophysiology** |
| --- | --- | --- | --- |
|  | **(n=212)** | **(n=165)** | **(n=47)** |
| **Animals and human studies included in the systematic review** | 33 (15.6%) | 24 (14.5%) | 9 (19.1%) |
| *Number of animal studies, median [Q1, Q3]* | 16.0 [11.00, 36.0] | 13.5 [10.25, 36.5] | 27.0 [19.0, 31.0] |
| *Number of human studies, median [Q1, Q3]* | 7.00 [6.00, 16.0] | 8.50 [5.50, 16.5] | 6.00 [6.00, 14.0] |
| *Separate meta-analyses for animal and human studies* | 18 (54.5%) | 13 (54.2%) | 5 (55.6%) |
| *Meta-analysis of only animal studies* | 9 (27.3%) | 7 (29.2%) | 2 (22.2%) |
| *Pooled meta-analysis of animal and human studies* | 6 (18.2%) | 4 (16.7%) | 2 (22.2%) |
| *Subgroup of animal and human studies* | 6 (18.2%) | 4 (16.7%) | 2 (22.2%) |

**Supplementary Table S3.** **Summary of results of the systematic reviews with meta-analyses.**

|  | **Overall** | **Therapeutic intervention** | **Pathophysiology** |
| --- | --- | --- | --- |
|  | **(n=212)** | **(n=165)** | **(n=47)** |
| **Systematic reviews** |  |  |  |
| **Number of included studies in the systematic review, median [Q1, Q3]** | 22.0 [13.00, 42] | 22.0 [12.0, 40.0] | 27.0 [15.0, 62.0] |
| **Earliest included publication date, median [Q1, Q3]** | 2002 [1994, 2007] | 2004 [1998, 2007] | 1993 [1978, 2002] |
| **Latest included publication date, median [Q1, Q3]** | 2017 [2016, 2018] | 2017 [2016, 2018] | 2017 [2015, 2018] |
| **Range of publication dates (years), median [Q1, Q3]** | 14.0 [10.0, 22.0] | 14.0 [10.0, 18.0] | 22.0 [14.5, 39.0] |
| **Studies included only in English** |  |  |  |
| Yes | 88 (41.5%) | 67 (40.6%) | 21 (44.7%) |
| No | 29 (13.7%) | 25 (15.2%) | 4 (8.5%) |
| Not reported | 95 (44.8%) | 73 (44.2%) | 22 (46.8%) |
| **Selection process** |  |  |  |
| *Reporting of number of screened studies* | 207 (97.6%) | 160 (97.0%) | 47 (100%) |
| *Reporting of number of excluded studies* | 199 (93.9%) | 155 (93.9%) | 44 (93.6%) |
| *Reporting of reason for exclusion* | 174 (82.1%) | 140 (84.8%) | 34 (72.3%) |
| **Description of studies** |  |  |  |
| *References of included studies* | 202 (95.3%) | 157 (95.2%) | 45 (95.7%) |
| *Reporting of year of publication (tables, figures)* | 192 (90.6%) | 148 (89.7%) | 44 (93.6%) |
| *Description of animal model (species and strain)* | 125 (59.0%) | 95 (57.6%) | 30 (63.8%) |
| *Definition of intervention* | 197 (92.9%) | 158 (95.8%) | 39 (83.0%) |
| *Definition of control* | 77 (36.3%) | 64 (38.8%) | 13 (27.7%) |
| *Number of animals in experimental and control groups* | 154 (72.6%) | 128 (77.6%) | 26 (55.3%) |
| **Meta-analyses** |  |  |  |
| **Number of included studies in the MA, median [Q1, Q3]*** | 9.00 [5.0, 20.0] | 9.00 [5.00, 20.0] | 10.0 [5.00, 22.5] |
| **Number of included experimental arms in the MA, median [Q1, Q3]*** | 13.0 [7.00, 33.5] | 13.0 [7.00, 30.25] | 19.0 [8.00, 50.5] |
| **Heterogeneity according to I^2^, median [Q1, Q3]*** | 77.0 [55.5, 90.7] | 77.95 [57.0, 91.0] | 74.0 [46.6 89.55] |
| **Meta-analyses with more than 10 experimental arms*** | 138 (65.0%) | 104 (63.0%) | 33 (70.2%) |
| *Funnel plot picture in the article* | 81 (58.7%) | 67 (64.4%) | 14 (42.4%) |
| *Reporting of visual inspection of funnel plot* | 62 (44.9%) | 54 (51.9%) | 8 (24.2%) |
| *Presence of small-study effect* | 49 (35.5%) | 38 (36.5%) | 11 (33.3%) |

MA: meta-analysis.

*For main outcome as defined by authors, or first reported outcome.

**Supplementary Table 4. Summary of results of meta-epidemiological analysis.**

|  | **Overall (n=63)** | | **Difference in SMD [95% CI]** | | **I^2^ (%)** | |
| --- | --- | --- | --- | --- | --- | --- |
|  | **N meta-analyses evaluating item** | **N meta-analyses with evaluable difference in SMD** | **REML meta-regression** | **RVE meta-regression** | **REML meta-regression** | **RVE meta-regression** |
| **Risk of bias** |  |  |  |  |  |  |
| Randomization | 63 | 37 | -0.04 [-0.32, 0.24] | 0.03 [-0.28, 0.33] | 54.1 | 77.4 |
| Group characteristics similar at baseline | 29 | 12 | -0.37 [-1.04, 0.29] | -0.23 [-0.76, 0.30] | 68.0 | 82.2 |
| Allocation concealment | 39 | 6 | - | - | - | - |
| Random housing of animals | 27 | 4 | - | - | - | - |
| Blinding of caregivers | 32 | 7 | - | - | - | - |
| Blinding of animal model | 10 | 3 | - | - | - | - |
| Random outcome assessment | 26 | 9 | - | - | - | - |
| Blinding of assessors | 56 | 24 | 0.39 [-0.18, 0.97] | 0.48 [-0.21, 1.18] | 67.8 | 95.8 |
| Attrition | 43 | 21 | -0.07 [-0.49, 0.36] | -0.04 [-0.79, 0.70] | 62.5 | 97.1 |
| Selective outcome reporting | 34 | 11 | -0.03 [-0.74, 0.67] | 0.06 [-1.14, 1.26] | 73.3 | 94.0 |

SMD: standardized mean difference; REML: restricted maximum likelihood estimator for meta-regression (standard meta-regression); RVE: robust variance estimator; 95% CI: 95% confidence interval

**Supplementary Figure S1. Two-step meta-epidemiological analysis [1]**. SMD: standardized mean difference; DSMD: difference in standardized mean difference.


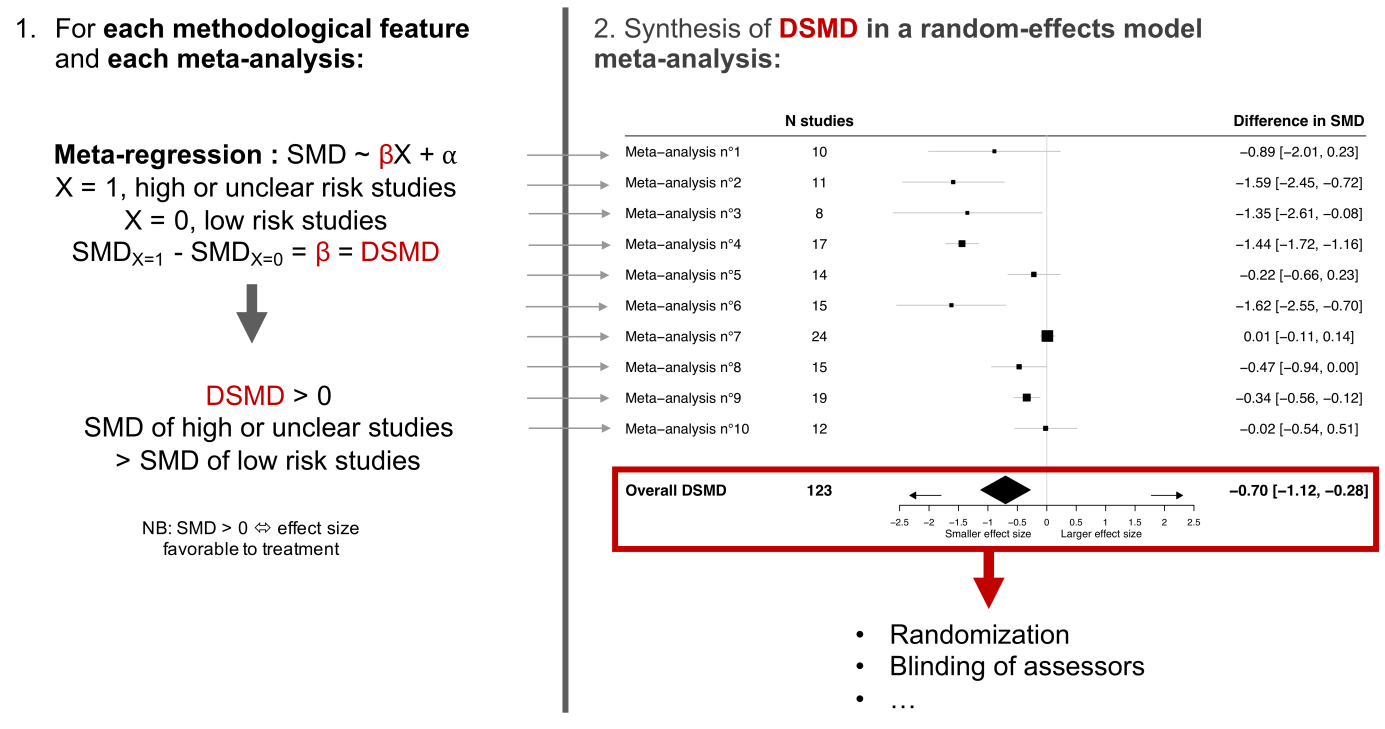


**Supplementary Figure S2.** **Selection process of studies included in the methodological review of included studies and the meta-epidemiological analysis**. Adapted from PRISMA flow diagram [2]. SMD: standardized mean difference.


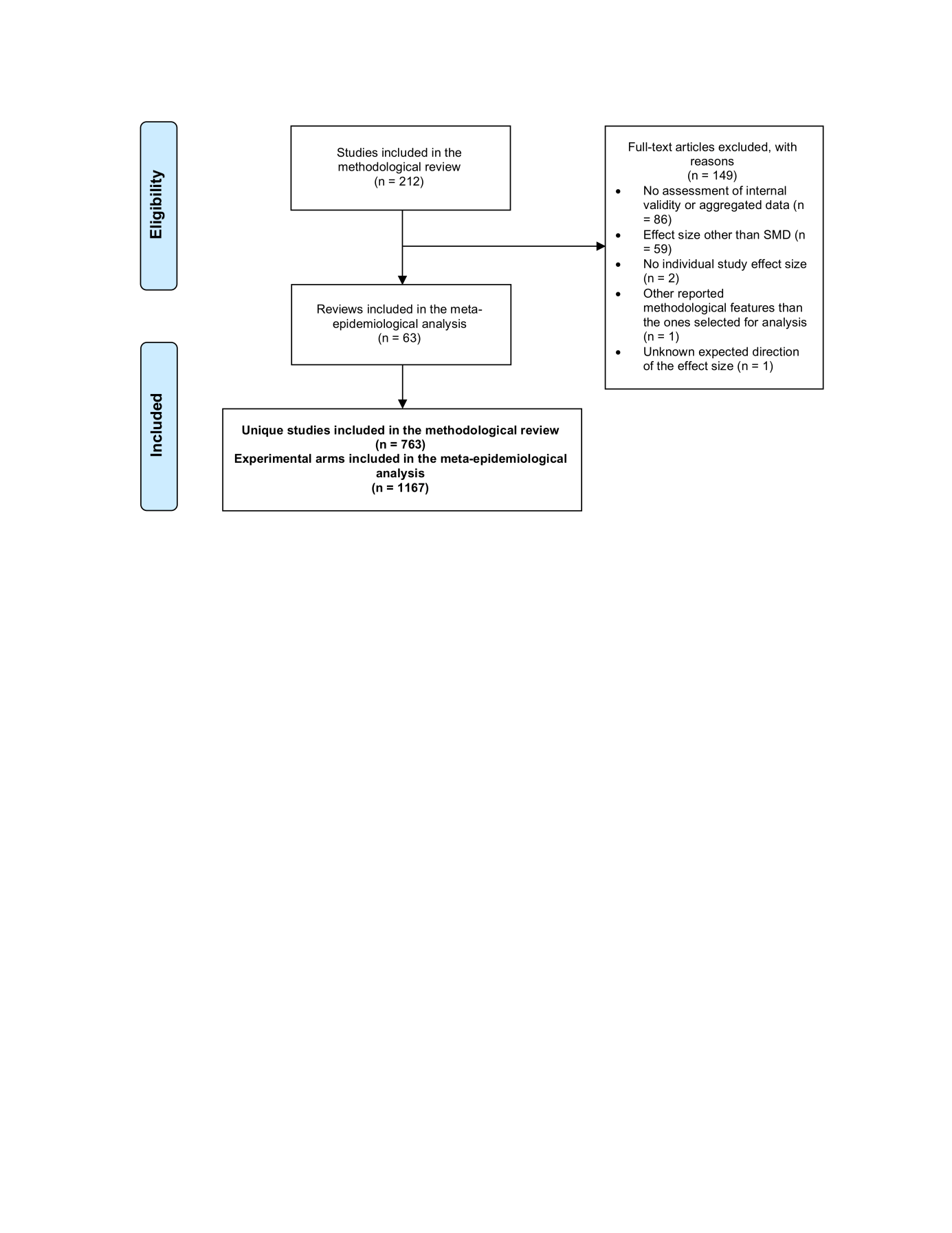


**Supplementary Figure S3. Difference in standardized mean difference (SMD) for risk of bias estimated by RVE meta-regression.** A positive difference in SMD reveals a larger effect size in studies at high or unclear risk of bias. A negative difference in SMD indicates a smaller effect size in case of threats to methodological quality. Het: heterogeneity; MA: meta-analyses; RVE: robust variance estimator.
